# The Effect of Health Information Technology on Health Care Provider Communication: A Mixed-Method Protocol

**DOI:** 10.2196/resprot.4463

**Published:** 2015-06-11

**Authors:** Milisa Manojlovich, Julia Adler-Milstein, Molly Harrod, Anne Sales, Timothy P Hofer, Sanjay Saint, Sarah L Krein

**Affiliations:** ^1^ University of Michigan School of Nursing Ann Arbor, MI United States; ^2^ University of Michigan School of Information Ann Arbor, MI United States; ^3^ Department of Veterans Affairs Center for Clinical Management Research Ann Arbor, MI United States; ^4^ University of Michigan Department of Internal Medicine Ann Arbor, MI United States

**Keywords:** interdisciplinary communication, health information technology, computer communication networks, hospital communication systems

## Abstract

**Background:**

Communication failures between physicians and nurses are one of the most common causes of adverse events for hospitalized patients, as well as a major root cause of all sentinel events. Communication technology (ie, the electronic medical record, computerized provider order entry, email, and pagers), which is a component of health information technology (HIT), may help reduce some communication failures but increase others because of an inadequate understanding of how communication technology is used. Increasing use of health information and communication technologies is likely to affect communication between nurses and physicians.

**Objective:**

The purpose of this study is to describe, in detail, how health information and communication technologies facilitate or hinder communication between nurses and physicians with the ultimate goal of identifying how we can optimize the use of these technologies to support effective communication. Effective communication is the process of developing shared understanding between communicators by establishing, testing, and maintaining relationships. Our theoretical model, based in communication and sociology theories, describes how health information and communication technologies affect communication through communication practices (ie, use of rich media; the location and availability of computers) and work relationships (ie, hierarchies and team stability). Therefore we seek to (1) identify the range of health information and communication technologies used in a national sample of medical-surgical acute care units, (2) describe communication practices and work relationships that may be influenced by health information and communication technologies in these same settings, and (3) explore how differences in health information and communication technologies, communication practices, and work relationships between physicians and nurses influence communication.

**Methods:**

This 4-year study uses a sequential mixed-methods design, beginning with a quantitative survey followed by a two-part qualitative phase. Survey results from aim 1 will provide a detailed assessment of health information and communication technologies in use and help identify sites with variation in health information and communication technologies for the qualitative phase of the study. In aim 2, we will conduct telephone interviews with hospital personnel in up to 8 hospitals to gather in-depth information about communication practices and work relationships on medical-surgical units. In aim 3, we will collect data in 4 hospitals (selected from telephone interview results) via observation, shadowing, focus groups, and artifacts to learn how health information and communication technologies, communication practices, and work relationships affect communication.

**Results:**

Results from aim 1 will be published in 2016. Results from aims 2 and 3 will be published in subsequent years.

**Conclusions:**

As the majority of US hospitals do not yet have HIT fully implemented, results from our study will inform future development and implementation of health information and communication technologies to support effective communication between nurses and physicians.

## Introduction

### Background

While more information and communication technology (ICT) will be deployed in the next 10 years than ever before, these developments do have risks to patients, leading some to call this a “dangerous decade” for health information technology (HIT) [1]. Poor communication between physicians and nurses is well known as one of the most common causes of adverse events for hospitalized patients [2-4] and a major root cause of all sentinel events [5]. HIT is often promoted as offering potential solutions to the problems uncovered by root cause analyses, including a variety of media that physicians and nurses are rapidly adopting to communicate with each other: the electronic medical record, computerized provider order entry, email, and pagers. While there is no doubt that increasing use of ICT will change the way nurses and physicians communicate, there is already evidence that communication technologies can contribute paradoxically to more [3,6], not fewer [7] communication difficulties. Thus, it is critically important to understand how communication technology is being used in health care and when it is most likely to achieve the goals of better communication and safer care [6].

The purpose of the study we outline here is to describe, in detail, the ways in which health information and communication technologies facilitate or hinder communication between nurses and physicians with the ultimate goal of identifying optimal ways to support effective communication. Effective communication is the process of developing shared understanding between communicators by establishing, testing, and maintaining relationships [8]. While this study is designed to provide generalizable lessons about the use of ICT in a rapidly changing health information technology environment, our description is designed to provide a framework and an exemplar for smaller investigations within single institutions that are seeking to understand and improve their communication culture.

As the use of newer communication technologies increases, physicians and nurses who previously came together frequently at the point of care delivery to discuss a patient face-to-face, are now increasingly separated by location and time and use a variety of technologies to transmit their discussions [9]. This change may improve the efficiency with which communication occurs but could also increase message ambiguity [6] and contribute to more adverse events [10], especially when complex situations arise [11]. Communication practices that consist of sending messages solely through a single medium, such as a pager, ignore the fact that a message sent via pager will differ from the same message sent verbally because content conforms to the medium in which it is presented [12].

### Theoretical Model

**Figure 1:**
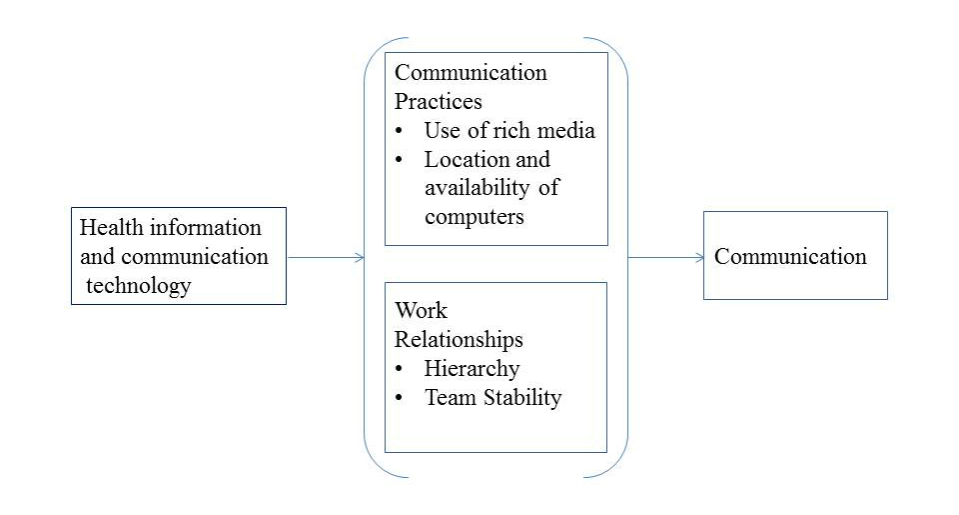
Theoretical model.

A theoretical framework allows researchers to empirically test relationships among concepts of interest, facilitating accumulation of knowledge and progression in the field. Our theoretical model provides a plausible explanation for both why and how technologies may influence communication. The theoretical model for this study explains how health information and communication technologies might meet the demand for more information through better alignment between technology and the message to be conveyed. Our theoretical model, depicted in Figure 1, posits that through communication practices and work relationships, health information and communication technologies contribute to alignment or mismatch between the technology and the message which, in turn, can affect communication.

Communication practices and work relationships form the context in which communication technology is situated. Communication practices are influenced by the use of rich media and the location and availability of computers. Media richness is defined as a characteristic of a communication medium that facilitates the ability of information being sent through that medium to change understanding [13]. Classification of media as rich or less rich is based on a medium’s capacity for immediate feedback, number of cues and channels used, personalization, and language variety [13]. Physician and nurse communication practices may or may not include a consideration of the richness of the media available to them. Media richness theory suggests that people should choose rich media such as face-to-face conversations or telephones to communicate about complex issues with lots of ambiguity. Rich media reduce ambiguity by enabling communicators to overcome different frames of reference and by providing the capacity to process complex messages. Less rich media provide fewer cues, restrict feedback, and tend to be impersonal but are effective for processing well understood messages and standard information [13]. Computer applications (eg, physician and nursing notes on electronic medical records (EMRs), computerized provider order entry (CPOE), electronic text) fall at the less rich end of the spectrum; computer applications are impersonal when they have very little opportunity to personalize the documentation or use a variety of language options.

The location and availability of computers influences communication practices by disrupting the development of distributed cognition [9,14], the notion that knowledge about a patient’s illness and treatment is distributed among the physicians and nurses (and other disciplines) who are providing care [15]. When physicians and nurses are dispersed to several far-flung locations to use communication technologies instead of being co-located, opportunities for sharing knowledge from differing perspectives are diminished [16], so the meaning of a message pertaining to the understanding of a situation is open to misinterpretation.

Work relationships affected by hierarchies within a health care team and team stability also influence how health information and communication technologies affect communication. Physicians and nurses must communicate with each other to solve patient care problems which require input from multiple disciplines for successful resolution [17]. Communication in these situations needs to facilitate consensus building that may be difficult to achieve for many reasons, but we have identified two in our theoretical model. First, the hierarchical nature of the relationship between physicians and nurses can hinder consensus building if nurses do not speak up about a patient care issue because they fear being embarrassed or censured by physicians [18]; nurses’ silence possibly contributes to adverse outcomes [19]. Thus, collaborative rather than hierarchical relationships are recommended to help to assure that all perspectives are brought to bear on a complex problem and arrive at consensus. Second, team stability may be especially relevant to the connection between communication technology and communication [20]. Team stability is defined as the same individuals who come together to work on collaborative tasks [20]. Team stability is important because it allows the development of relationships necessary to facilitate understanding of varying perspectives [21]. Individuals who communicate more with each other become more similar as they increasingly share their beliefs and knowledge [22]. Stable physician presence on the health care team makes it easier for clinicians to find common ground (shared knowledge between two communicators [23]) and construct a shared reality [24].

## Methods

### Study Overview

We will use a sequential, mixed-methods study design [25], beginning with a quantitative survey and using those results to inform a two-part subsequent qualitative phase [25]. Mixed-methods blend the strengths of both qualitative and quantitative research, generating additional data and enhancing insights to provide a robust view of the phenomenon under investigation. We will integrate multiple forms of data, mixing methods through an approach known as connecting data, which involves using information gained through one method to inform subsequent data collection using another method [25]. Our purpose in using mixed-methods is to provide a contextualization of communication technologies being used in medical-surgical units [25], and to develop a more complete understanding of how health information and communication technologies facilitate or inhibit understanding between physicians and nurses when they communicate. Information gained through the quantitative survey will inform subsequent data collection using qualitative methods. Specifically, stratified purposeful sampling will be applied to results from the surveys to identify sites that vary in their use of communication technologies for the qualitative phase of the project. Textbox 1 provides an overview of our study design.

**Overview of study design.**

Design

- Quantitative phase
  - Administer survey to Chief Nurse Executives, or other designated personnel
  - Use Healthcare Information and Management Systems Society's (HIMSS) model to categorize survey results
  - Analyze survey data
- Qualitative phase
  - Part 1
    - Select cases from the survey for semi-structured interviews based on maximal variation in communication technologies
    - Collect telephone interview data from 8 hospitals
  - Part 2
    - Select hospitals for intensive investigation based on information about communication practices and work relationships gained from telephone interview data
    - Collect data using ethnographic methods in 3-4 hospitals
    - Analyze data from parts 1 & 2 using thematic techniques

### Aim 1

The first aim of our study is to identify the range of health information and communication technologies used in medical-surgical units.

#### Study Sample

The National Nursing Practice Network (NNPN) will be used as a sampling frame for this study. The NNPN is a consortium of 108 acute care agencies committed to the use of evidence-based practices. The majority of member organizations in NNPN are in the Midwest, but there are NNPN member organizations in 29 contiguous states, Hawaii, Alaska, and Puerto Rico. NNPN facilities range from academic medical centers in urban areas to small rural hospitals, single hospitals, and multi-hospital systems. Almost one quarter of NNPN members are part of the Veterans Health Administration, the largest integrated health system in the United States [26] and the health system with the most mature electronic medical record [27]. Given the wide range of adoption of electronic medical records nationally [28], we expect that the use of communication technologies within hospitals across our sample will also vary widely.

#### Survey Development

Questions asked in the American Hospital Association annual hospital survey’s Information Technology supplement (AHA IT) related to communication technology (electronic clinical documentation, CPOE, decision support) were used as the starting point for our survey. The AHA IT supplement identifies both minimum functionalities hospitals need to label a system an electronic records system as well as comprehensive functionalities considered advanced with regard to electronic records systems [29]. While the AHA IT supplement asks questions about some electronic functionality related to communication, such as clinical documentation (ie, physician progress and discharge notes, nursing assessments) and order entry (ie, CPOE), it does not ask about other functionalities, such as characteristics of the paging system in use or computer-mediated communication between physicians and nurses. We added questions about these other functionalities (ie, pagers, computer-mediated communication devices such as email, cellular phones, tablets, electronic white boards) and grouped them according to categories of media richness. Information gathered through the AHA IT supplement is not available the same year it is collected, creating a lag time and possibly out-of-date information. Thus, we included key AHA IT questions to gather the most current information about communication technology through our survey. In pilot testing the preliminary survey took 10-20 minutes to complete. The survey will be administered via the web to contain costs and increase the efficiency and accuracy of data collection. We will follow recommendations for web survey visual appearance [30].

#### Data Collection

##### Procedures

We will follow web survey implementation procedures as outlined by Dillman and colleagues [30] to increase response rates and use a multi-mode survey method. The Chief Nurse Executives (CNEs) of all 108 members of the NNPN will be invited to participate in the study. We will send a postal letter of invitation to the CNE of each hospital at his/her institutional affiliation address since contact via post is associated with higher response rates [30,31]. The postal mail letter will introduce the study and include a survey link with a personal access code that will be assigned to each respondent. Assigning a unique identification number or personal access code allows the respondent to complete the survey without further contact, prohibits the same respondent from completing the survey more than once, and allows us to link a particular survey with a specific hospital. The postal mail letter will serve as the first of three contacts, since multiple contacts using different modes is the most effective method of increasing response rates, and does not increase measurement error [30]. We will include a “fact sheet” highlighting key study strengths and advantages to participation as well as a $20 gift card in the letter as an advance incentive to complete the survey. Advance financial tokens are one of the largest contributors to improved response rates [30,32], but have limited success when sent electronically. The CNE will be instructed to work with an informatician, physician, or other appropriate personnel as needed to complete the survey. Thus, we will not know who completed the survey, which helps to protect the anonymity of responses.

Next, we will send an email reminder to identified respondents using email addresses provided by the NNPN. Email requests to complete the online survey will be sent within a week of sending the postal mail letter, although the optimal timing sequence for web surveys has not been determined [30]. In this email reminder we will resend the “fact sheet” in case the earlier mailing was misplaced. Using a combination of postal and email contacts has been associated with response rates of ≥50% [30]. Then we will send out a second email reminder about 1 week later, varying the message and its appearance, which has also been shown to increase response rates. Because of the success we [33,34] and others [35,36] have had with the Dillman method, we anticipate a response rate of >50%, sufficient for the purposes of this study.

##### Remaining Data Needs

The survey will ask questions to identify the use of hospitalists on medical-surgical units and hospital characteristics (ie, size, urban/rural location, teaching status) because certain characteristics are associated with more or less adoption of technology. This information will allow us to stratify our sample for aim 2.

#### Data Analysis Plan

The analytic plan for aim 1 will be quantitative, using descriptive statistics to characterize the range of health information and communication technologies used at participating hospitals. We will stratify survey responses into two broad categories of hospitals, those that have few and those that have more health information and communication technologies based on their location on the Healthcare Information and Management Systems Society’s (HIMSS) electronic medical record (EMR) adoption model. The primary unit of analysis will be the hospital unit. Since aim 1 is descriptive, we did not conduct a power analysis.

We will use descriptive statistics to describe the distribution, dispersion, and central tendency of each concept in our survey. We will calculate ranges and mean values for certain concepts. For example, it will be possible to determine that on average 65% of physicians and nurses frequently engage in face-to-face communication (more than once a week). This information will provide the context for understanding communication practices and work relationships in aims 2 and 3.

Using media richness categories to stratify our sample into hospitals that have more or less health information and communication technologies will not be possible since technology is only classified on the media richness spectrum. We will use the HIMSS model to stratify survey responses into two broad categories of hospitals that have more or less technologies. The HIMSS model uses an 8-step scale (0-7) to identify hospitals’ trajectories towards a fully paperless environment, which is stage 7 [28]. Hospitals below stage 4 will be considered to have a low likelihood of many health information and communication technologies while hospitals at stage 4 or above will be considered to have a high likelihood of these technologies. Stage 4 will be the cutoff because physicians and nurses communicate through CPOE, and miscommunication through CPOE is associated with increased errors [6]. The most recent data (fourth quarter, 2014) indicate that 68% of 5467 surveyed hospitals are at stage 4 or above [28], thus from the entire NNPN network of >100 hospitals there will be sufficient variation in HIT needed for this study. Table 1 compares information on technology functions as identified through the study survey (based on questions from the AHA IT supplement) with the HIMSS model [28].

Once our sample has been stratified into low and high health information and communication technologies, we will describe each stratum, guided by our theoretical model. The 2 groups, high and low communication technology hospitals, will be used as the starting point for aim 2.

**Table 1:** A comparison of survey information and the HIMSS EMR adoption model.

Survey information (from AHA IT supplement)	Corresponding placement on HIMSS model	EMR adoption model
	Stage 0	All 3 ancillaries not installed
Results viewing	Stage 1	Ancillaries (lab, radiology, pharmacy) installed
	Stage 2	Clinical data registries (CDR), controlled medical vocabulary, clinical decision support (CDS), may have document imaging; health information exchange capable
Clinical decision support (CDS): error checking only	Stage 3	Nursing/clinical documentation (flow sheets), clinical decision support (error checking)
CDS: error checking and clinical protocols	Stage 4	CPOE, clinical decision support (clinical protocols and error checking)
Computerized provider order entry	Stage 4	
	Stage 5	Closed loop medication administration
Electronic clinical documentation	Stage 6	Physician documentation (structured templates), full CDS (variance & compliance), full radiology picture archiving and communication system (R-PACS)
	Stage 7	Complete EMR; continuity of care document (CCD) transactions to share data; data warehousing; data continuity with emergency departments (EDs), ambulatory, outpatient (OP)

### Aim 2

The second aim is to describe communication practices and work relationships between physicians and nurses that may be influenced by health information and communication technologies in medical-surgical acute care units.

#### Sampling and Study Setting

**Figure 2:**
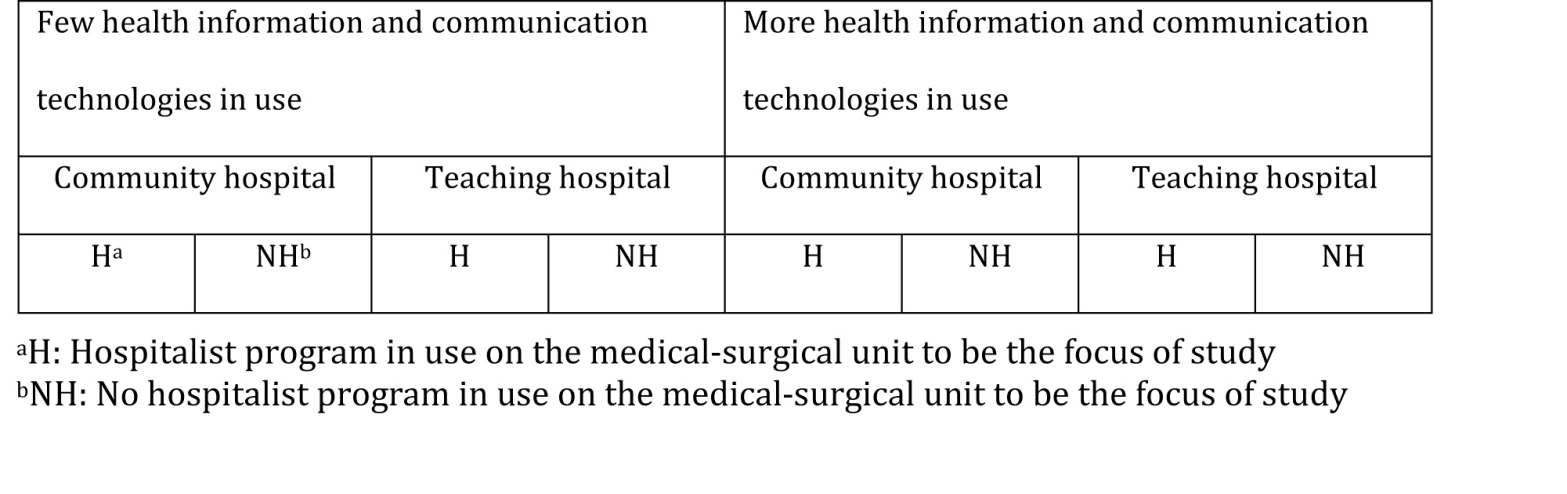
Sampling plan for aim 2.

We will use the results of aim 1 to divide a sub-sample of hospitals into 2 groups. We will use purposive sampling [37] based on high/low use of health information and communication technologies, and stratify by hospital type and the use of hospitalist physicians on medical-surgical units. Each case will represent a prespecified combination of concepts thought to influence communication practices and work relationships [19,38,39]. Answers to demographic-type questions on the survey will allow us to sort surveys into 1 of 8 categories (bottom row in Figure 2) [37]. Our goal will be to recruit informants from 8 hospitals for telephone interviews.

#### Recruitment

Our recruitment will begin with the CNE using a snowball sampling technique to recruit up to 3 informants at each participating hospital.

#### Data Collection: Telephone Interviews

A semi-structured interview guide will be developed by the research team based on our conceptual model and insights gained from pilot studies (on the topic of communication between physicians and nurses) and survey results. The guide will focus on understanding communication practices and work relationships in each medical-surgical unit, and how these have influenced the health information and communication technologies in use. The guide will also be designed to encourage ideas or concepts not included in our conceptual model to surface. Interviewees will be asked to validate survey information on technologies in use on medical-surgical units, in case new technologies were deployed since the survey was completed. Telephone interviews will continue until informational redundancy has been reached at each facility (ie, no new information is being collected) or all potential participants have been exhausted. We anticipate that each interview will last 30-40 minutes.

#### Data Analysis Plan

The analytic approach for aim 2 will consist of qualitative and quantitative components. First we will qualitatively analyze telephone interview transcripts using a directed content analysis approach [40], and approach the data with analytic categories (ie, codes) derived from our theoretical model, also looking for other analytically relevant categories in the data. Once qualitative analysis is complete we will triangulate qualitative results with survey results. For example, through triangulation we will be able to compare communication practices and work relationships (qualitative data from telephone interviews) between a medical-surgical unit in a hospital that reported using only paper-based patient medical records on the survey with communication practices and work relationships in a medical-surgical unit in another hospital where patient medical records are 100% computer-based. Through triangulation we will begin to understand potential relationships between variation in health information and communication technologies, communication practices, and work relationships to inform our choice of sites for ethnographic study.

### Aim 3

The third aim will explore how differences in health information and communication technologies, communication practices, and work relationships between physicians and nurses influence communication.

#### Sampling and Study Setting

Our theoretical model and emerging themes generated through analysis of telephone interviews will guide our choice of hospitals. We will purposefully choose up to 4 hospitals from the sub-sample of 8 hospitals that provided informants for telephone interviews.

#### Data Collection

Hospital visits will be sequential to allow time for data analysis between site visits, so that we can use knowledge gained from one hospital to inform our approach to subsequent site visits in other hospitals.

##### Direct Non-Participant Observation

To “kick off” the ethnographic portion of the study, two research team members will travel to each hospital to meet with key personnel (ie, Chief Nurse Officer, Chief Medical Officer, nurse manager, and medical director of medical-surgical unit). The purpose of the kick-off is to garner support for the study, discuss logistical issues, provide entrée for research assistants, and establish buy-in. During meetings we will gather information on the roles and responsibilities of nurses and physicians, which will be validated when research assistants conduct general observation. We will establish buy-in by soliciting input from key personnel, to engage them more thoroughly and demonstrate shared ownership of the project, important strategies for success [41,42].

Immediately after the kick-off, a pair of trained research assistants will be deployed to the medical-surgical unit selected for observation and engage in direct non-participant observation. We anticipate that research assistants will be at each site for about 2 weeks. Using the procedure most acceptable to all, the research assistants will observe physicians and nurses in the hallway, nursing station, physician team room, and public spaces on each unit, to unobtrusively observe communication events between physicians and nurses as they occur in these spaces. Research assistants will observe the general flow of work and document their observations of health information and communication technologies, including the use of rich as well as less rich media for communication purposes.

Observations will be conducted in 4-8 hour blocks of time during different shifts but weighted primarily towards the hours of 07:00-20:00 which is when the bulk of patient activities (and communication between physicians and nurses about patients) occur. Through observation we will capture different situations and different communication uses in those situations. Research assistants will also use informal interview techniques in situations where they do not understand a certain practice, allowing us to better describe communication practices that are in use. All observations will be captured in field notes. De-identified field notes and summaries will be entered into NVivo and coded using codes that we developed in aim 2. New emerging codes will be included as well.

##### Shadowing

Research assistants will shadow nurses and physicians in 4-hour blocks of time on the medical-surgical unit. We will recruit up to 7 nurses and 7 physicians to be shadowed, purposively recruiting nurses with a variety of experience, and physicians at varying stages of their career (eg, intern, 3rd year resident), and different specialties (eg, internist, general surgeon). Research assistants will shadow physicians and nurses independently. These specific observations will center on each individual’s daily interactions with all paper, computer, pager, telephone, and oral patient-related communications and record keeping activities. During shadowing we will gain deeper understanding about how physician roles and responsibilities influence communication with nurses.

To better understand why physicians and nurses use a specific health information or communication technology (eg, CPOE, pager, email), as part of their communication practice we will use a specific interview technique known as *think-aloud* in combination with shadowing. The think-aloud technique takes place during the course of action and involves asking participants what they are thinking and feeling as they communicate about patient care. This method provides the meanings behind the actions that are otherwise difficult to obtain, and will give us greater insight into communication practices and work relationships. Research assistants will record the length of conversations between physicians and nurses, and will take field notes during the shadowing experience, to be treated the same as observation field notes and summaries described above.

##### Focus Groups

The purpose of gathering data through focus groups is to provide validation for what we observe and to develop greater understanding of communication practices and work relationships from the perspective of physicians and nurses. Focus group questions and probes will be identical for both groups, having been used in a pilot study where we found that physicians and nurses described how a specific technology influenced the content of the message. We will invite up to 9 nurses to participate in one focus group (about 3 from each shift) and a similar number of physicians to participate in a separate focus group to better understand communication and organizational issues from each group and derive greater understanding of differences between the groups [43]. Nurse characteristics such as age, experience, and shift most often worked have been significantly linked to communication in previous studies [44]. Physician characteristics such as specialty and level (ie, attending, fellow, resident) may influence communication with nurses and physician attitudes towards hierarchical differences [45]. Focus groups will be audio recorded and transcribed verbatim.

##### Artifacts

Artifacts are a key source of data in ethnographic studies [46] and are useful organizational resources which provide guidance and support in the conduct of work [47]. Research assistants will collect documents such as policies and procedures (eg, policies on processing STAT orders), incident reports, and any other documents related to health information and communication technologies, communication practices, or work relationships. We will use artifacts to compare formal guidelines for communication with communication practices that actually occur, and to determine from incident reports if communication or technologies were at the root cause of the event. Deployment of health information and communication technologies are regulated by policies and procedures but frequently not useful in clinical practice, leading to workarounds with potentially deleterious consequences for patients [48]. We will abstract corresponding physician and nursing notes from a small sample of patient medical records to determine concordance on the plan of care. Absence of similar themes between physician and nursing notes would suggest lack of shared understanding and ineffective communication. We will conduct preliminary data analysis after observation, shadowing, and focus groups have been completed at each site.

##### Follow-Up Site Visits

Follow-up site visits will be conducted as a form of member checking. We will travel to each site to present findings and have key personnel put findings into context. We will ask questions about observed processes or acquired artifacts that may lead to additional insights about communication practices and work relationships at each hospital. For example, we may observe variable understanding in a policy regarding how to communicate STAT orders, or implement a bundle to prevent health care associated infections, but not understand the genesis of that variability until we talk with key leaders.

#### Data Analysis Plan

The data analysis plan for aim 3 will be similar to and build on analysis done to meet aim 2. We will use directed content analysis on all data gathered through aim 3 to look for themes generated earlier as well as themes consistent with concepts in our theoretical model. We will then combine qualitative data from aims 2 and 3 using content analysis and merge the qualitative data set with the quantitative data set from aim 1 to triangulate the data and conduct final data analysis of the study. Developed codes and themes from aim 2 will be used in aim 3 to better draw out the key commonalities and differences across the study sites. We will explore common and divergent themes [49] by asking questions consistent with our theoretical model such as (1) how did communication practices contribute to patient event X only in this hospital, although all hospitals used the same communication technology? and (2) how did work relationships lead to hospitals coming up with such different policies related to the same health information technology process (ie, process for STAT orders)? Once the qualitative data have been fully analyzed, the two datasets (quantitative and qualitative) will be merged using concurrent data analysis. This requires triangulating the data to develop a more complete picture of the findings [50]. This approach ensures that the study aims are fully explored and all findings are weighted equally.

## Results

This 4-year study is well-aligned with the Agency for Healthcare Research and Quality’s (AHRQ) interest in the nature of clinical expertise in individual and team decision making. Health care work is information sensitive [8], and information must be understood before it can be acted upon [51]. Study results will provide a rich understanding of the many factors that influence communication so that strategies to promote improvements can be developed and tested. Current health information and communication technologies do not facilitate knowledge building required to solve complex patient care problems, nor do we know the best way to configure them to improve their functionality. Work relationships and communication practices influence what technologies will be used, and may offer insights into how to improve the use of health information and communication technologies, but have rarely been studied in context.

## Discussion

The ability to capture a range of contexts in which communication occurs is a strength of our methods and this study. Authentic work relationships are visible only through direct observation, semi-structured interviews, and focus groups. Comparing face-to-face with technology-mediated communication will provide insight into how clinical meanings are negotiated for mutual understanding and agreement on action. Insights into the various communication practices will illuminate what types of information get communicated, how and when they are communicated, and what the resulting activities are because of these exchanges. This study will also provide insight and reason into how miscommunication can occur and possible ways to improve communication so that patient care is not adversely affected. Our approach is deliberately open-ended so that non-verbal communication events which are not well-captured in quantitative surveys will be more accessible and documentable. These “hidden” communications are necessary to understand the full scope of communication practices and to capitalize on those processes that work and those that need improvement.

There are several challenges associated with this project. The first challenge will be to get hospitals to agree to participate in the survey portion of the study. We will use the Dillman approach and provide advance incentives to increase study participation as described in the methods for aim 1. A related challenge will be to get physicians and nurses to participate in observation and shadowing. We will post flyers explaining the study and inviting participation in each unit. In the nurse/physician communication pilot study we found that having the support of the physician director of the unit was helpful in enlisting physician participation. Nurses and physicians may have to come in on a day off or take time away from direct patient care; each nurse and physician will receive a $40 gift card as a token of appreciation. The introduction of observers could be uncomfortable for some individuals. We will use strategic integration to gradually acclimatize study participants to observer/research assistant presence. We will not observe any potential subject who prefers not to participate. Observations might interfere with the orderly conduct of patient care. This risk will be offset by having unobtrusive, well-trained observers. No identifying information on staff will be gathered.

In summary, a fuller understanding of clinical work in context is essential if interventions aimed at improving interdisciplinary communication and using technology to do so will be realized. This study will identify those health information and communication technologies that support mutual understanding between nurses and physicians and those that are more prone to misunderstanding, so that prior communication failures do not haunt future communication strategies which in the 21st century will depend heavily on technology.

## Abbreviations

AHA IT: American Hospital Association Information Technology
CNE: Chief Nurse Executives
CPOE: computerized provider order entry
EMR: electronic medical record
HIMSS: Healthcare Information and Management Systems Society
HIT: health information technology
ICT: information and communication technology
NNPN: National Nursing Practice Network

## Appendix

Multimedia Appendix 1 NIH Review summary statement.
