# Supplementary material for: The Effect of Health Information Technology on Health Care Provider Communication: A Mixed-Method Protocol
Source: JMIR Res Protoc. 2015 Jun 11;4(2):e72. doi: 10.2196/resprot.4463 (PMC4526935; doi:10.2196/resprot.4463)
Supplement: Multimedia Appendix 1 [file resprot_v4i2e72_app1.PDF]

SUMMARY STATEMENT
(Privileged Communication)

PROGRAM CONTACT:
Angela Nunley
(301) 427-1505
angela.nunley@ahrq.hhs.gov

Release Date: **11/07/2013**

Application Number: **1 R01 HS022305-01A1**

Principal Investigator

MANOJLOVICH, MILISA PHD

Applicant Organization: UNIVERSITY OF MICHIGAN

Review Group: **HQER**
Health Care Quality and Effectiveness Research

Meeting Date: **10/28/2013**
Council: **JAN 2014**
Requested Start: **04/01/2014**
RFA/PA: **PA11-198**
PCC: **CP3**

Project Title: **The Effect of Health Information Technology on Healthcare Provider Communication**
SRG Action: **Impact Score: 25 Percentile: 8**

Human Subjects: **30-Human subjects involved - Certified, no SRG concerns**
Animal Subjects: **10-No live vertebrate animals involved for competing appl.**
Gender: **1A-Both genders, scientifically acceptable**
Minority: **1A-Minorities and non-minorities, scientifically acceptable**
Clinical Research - not NIH-defined Phase III Trial

Project Year	Direct Costs Requested	Estimated Total Cost
1	224,692	348,761
2	227,883	353,714
3	308,658	479,091
4	280,593	435,529
TOTAL	1,041,826	1,617,094

ADMINISTRATIVE BUDGET NOTE: The budget shown is the requested budget and has not been adjusted to reflect any recommendations made by reviewers. If an award is planned, the costs will be calculated by Agency grants management staff based on the recommendations outlined below in the COMMITTEE BUDGET RECOMMENDATIONS section.

RESUME AND SUMMARY OF DISCUSSION: This resubmission R01 grant application entitled *the Effect of Health Information Technology on Healthcare Provider Communication* is in response to PA-11-198 “Understanding Clinical Information Needs and Health Care Decision Making Processes in the Context of Health Information Technology (IT) (R01)” from the University of Michigan by Milisa Manojlovich, PhD, Principal Investigator (PI). This 4-year grant application is to understand how health information technologies facilitate communication between nurses and physicians.

The investigators have appropriately responded to previous reviewers’ concerns by reducing the scope of the project and focusing on medical errors.

Additional strengths are identified in this application. Exploring the use of information technology by health care providers is a significant area of research; the integration of nurses and physicians to the focus groups is a very innovative approach; and potential pitfalls are addressed. Additionally, the conceptual framework is well articulated with the proposed aims of the study; the ethnographic methods for gathering data for analysis are well documented; and the categorization of using the Healthcare Information and Management Systems Society’s (HIMSS)\Electronic Health Records (EHR) Adoption model is an excellent way to systematically assess a given institutions state of HIT adoption. The sampling strategy for Aim 2 is reasonable; the environment is robust; letter of support are provided; and the investigators are very strong, particularly in the field of qualitative methods and nursing care.

However, weaknesses are identified. More emphasis should be paid to the inclusion of additional data collection and telephone interview, and the dissemination plan should be enhanced by including an institutional repository.

Overall, improving the knowledge of interdisciplinary communication by using information technology is an important component for optimal health care . Based on all these factors, the reviewers rated this application Outstanding/Excellent and recommended it for further consideration with a level of “High Enthusiasm.”

DESCRIPTION (provided by applicant): The purpose of this study is to describe in detail how communication technologies facilitate or hinder communication between nurses and physicians with the ultimate goal of identifying the optimal ways to support effective communication. Communication failures between physicians and nurses are one of the most common causes of adverse events for hospitalized patients as well as a major root cause of all sentinel events. Communication technology (i.e., the electronic medical record, computerized provider order entry, email, and pagers), created through health information technology (HIT), may help reduce some communication failures but increase others because of an inadequate understanding of how communication technology is used. Our theoretical model is based in communication and sociology theories to describe how communication technologies affect communication through communication practices (i.e., use of rich media; the location and availability of computers) and work relationships (i.e., hierarchies and team stability). Specific Aims: We seek to: (1) identify the range of communication technologies used in a national sample of medical-surgical acute care units; (2) describe communication practices and work relationships that may be influenced by communication technologies in these same settings; and (3) explore how differences in communication technologies, communication practices, and work relationships between physicians and nurses influence communication. Design and Methods: This study will use a sequential mixed methods design, beginning with a quantitative survey followed by a two-part qualitative phase. Survey results from Aim 1 will provide a detailed assessment of communication technologies in use (important for meeting meaningful use criteria) and help identify sites with variation in communication technologies for the qualitative phase of the study. In Aim 2 we will conduct telephone interviews with hospital personnel in up to 8 hospitals to gather in depth information about communication practices and work relationships on medical-surgical units. In Aim 3

we will collect data in 4 hospitals (selected from telephone interview results) via observation, shadowing, focus groups, and artifacts to learn how communication technologies, communication practices, and work relationships affect communication. Significance: Current communication technologies are based on models of individual decision-making and may not be sufficient for, or may interfere with, decisions requiring input from multiple perspectives. Complex one-of-a-kind problems, such as those frequently encountered in hospital settings, require knowledge building as part of the problem solution, which current communication technologies do not facilitate. As the majority of American hospitals do not yet have HIT fully implemented results from our study may inform future development and implementation of communication technologies.

PUBLIC HEALTH RELEVANCE: The Effect of Health Information Technology on Healthcare Provider Communication Project Narrative The purpose of this study is to describe in detail how communication technologies make it easier or more difficult for nurses and physicians to communicate with each other, with the ultimate goal of finding ways to support effective communication. As technology becomes more common in hospitals, ways to improve communication between nurses and physicians are urgently needed because poor communication between physicians and nurses has long been a leading cause of harm to hospitalized patients.

CRITIQUE NOTE: The sections that follow are the essentially unedited, verbatim comments of the individual committee members assigned to review this application. The attached commentaries may not necessarily reflect the position of the reviewers at the close of group discussion, nor the final majority opinion of the group. The above RESUME/SUMMARY OF DISCUSSION represents the evaluation of the application by the entire committee.”

CRITIQUE 1:

Significance:	4
Investigator(s):	2
Innovation:	2
Approach:	5
Environment:	3

Overall Impact:
Strengths:
- Solid, extensive plan to isolate some specific, vital communication aspects in healthcare delivery.
- Data from multiple sites boosts the limits of in-depth analysis, which is usually fairly limited.
- Well-poised ethnography.

Weaknesses:
- There could be a plan to establish buy-in at each recruited hospital: have hops somehow share ownership of the project so that the support and confidence can be transmitted to the eventual nurse and physician participants and to the others who will be in the presence of the ethnographically based shadowing.
- The revised proposal notes letters of support from hospitals. Possibly a weakness not to orient communication analysis more specifically on medical errors – that is noted in the beginning, but is not sustained, not noted as a leading value to be gained.
- The proposal overall covers a great range, which is concerning – there are too many contexts affecting communication when the range is so vast. The proposal has been reduced and refined in scope to some degree by focusing on medical errors – which it originally noted as an impetus for improvement – but this declared focus has not quite convincingly been engineered into the study.

1. Significance:

Strengths:

- The case is made fairly well that communication problems between physicians and nurses are a significant contributor to problems in healthcare, such as medical errors.
- This study ought to yield information regarding what is happening in healthcare delivery communication as settings transition from face-to-face, deep communication to a mix including a lot of e-communication. The couple examples –STAT orders, etc. – are realistic.

Weaknesses:

- While this is a limit of open-ended qualitative research, a weakness here is that it is not known that findings will necessarily lead to communication improvements in healthcare.

2. Investigators:

Strengths:

- Very strong team, with strength in qualitative methods as well as nursing care, and the practice of medicine.

Weaknesses:

- No strong weaknesses. Dependence upon research assistants is a weakness – their task is a very sensitive one, and it will be crucial to “get it right” at the shadowing and think-out-loud steps.
- **Update - some methodological strengthening in this regard has been added. **

3. Innovation:

Strengths:

- Innovative: unique qualitative methods, strong theoretical background to isolate a discrete range of communication modes, and physician/nurse communication.
- Good to do some purposive sampling via a nurses practice network.

Weaknesses:

- No notable weaknesses.

4. Approach:

Strengths:

- Phases are well-thought out. The quantitative survey at the beginning does seem to inform the next phase of in-depth ethnography.
- Ethnographic methods for gathering data and for analysis are strong.

Weaknesses:

- Rather than selecting hospitals based on being at extremes of the communications profile spectrum, hospitals could be selected by being not-quite at the extremes, and not in the middle. In example, hospitals in the second and fourth quartiles could be used. This would achieve the goal of having some spread or difference between the two types of hospitals, but avoid a pitfall of analyzing quite unusual hospitals. At the 2nd and 4th quintiles, hospitals would be quite generalizable, while they almost likely will not be very representative of hospitals generally if selected due to being extremes.

- It is unclear how the necessity of the first phase, the survey – It is unclear what is the value of having those data for the eventual study goals, other than to help determine which hospitals to investigate in depth It is unclear how the data will be valuable, and should be the topic of a valuable publication.
- This study takes a more fundamental, formative approach than a series of root cause analyses, but might benefit from actually analyzing some problems and failure in depth.

- Recording conversations in the actual course of medical care delivery may not be feasible when considering one aspect: patient names and info will be “gathered” by the recording, and patients will have no awareness that their names are being gathered into a data set, and they will have no opportunity to consent or decline having their name / health status be entered into the data set of a study. This effort is “research,” versus regular delivery of care or being regular quality improvement. This aspect discussed or “cleared” by some IRB-type evaluation is needed. It might be fitting to drop the recording, or to record in some limited, well-prescribed circumstances.
- **Update: selection of hospitals has been developed, specifically by sampling from each of several levels of health IT adoption.
- Much of the proposal remains expansive, vague, and without clear methodology and purpose. It is difficult to see the value in resulting data.
- It still seems that audio-recordings will be done regarding specific patient care episodes, and patient names may be recorded in these audio recordings. It seems to remain that patient-specific information, including names along with that person’s medical conditions and other personal information. It seems to continue to be the case that patients will not know that their info is being gathered, even if it is to be deleted to some degree when transcribed.**

5. Environment:

Strengths:

- Very favorable.

Weaknesses:

- None noted

Degree of responsiveness: Responsive

Strengths:

- Responsive

Weaknesses:

- None noted

Budget and Period of Support: Fine

Strengths:

- Fine.

Weaknesses:

- None noted

Protection of Human Subjects from Research Risk: Overall, fine. Well-developed

Strengths:

- Overall, fine. Well-developed.

Weaknesses:

- The audio recording to be done of providers in the process of healthcare delivery will almost surely capture patient names, and the patients will have no idea their name or case is becoming part of a research data set, and so will have no way to make informed consent. I would suggest eliminating or re-designing the audio recording, or at least having some sort of conference with an IRB to double-check this.

Inclusion of Women and Minority Subjects: Fine

Strengths:

- Fine. Includes women, since females are well-represented in nursing healthcare.
- Inclusion of minorities according to workforce characteristics is noted.

Weaknesses:

- None noted

Inclusion of AHRQ Priority Populations:
Strengths:
- Med/surg = chronic illness, elderly, disabled.

Weaknesses:
- None noted

Resource Sharing Plan(s):
Strengths:
- Dissemination plan is in place – seems fine.
- Each year is under $500,000, so no need for plan to share data set.

Weaknesses:
- None noted

CRITIQUE 2:

Significance:	2
Investigator(s):	1
Innovation:	2
Approach:	2
Environment:	1

Overall Impact:
Strengths

- The topic is significant in that it purports to addresses the types and issues with communication between physicians and nurses commonly used in the setting of an electronic health care environment. The investigators state that a "fuller understanding of clinical work in context is essential if interventions aimed at improving interdisciplinary communication and using communication technologies to do so will be realized." The conceptual framework is well articulated with the proposed aims of the study. The richness of the research environment and group of investigators has a high likely hood to the success of the project. Aspects of the previous reviews are sufficiently addressed.

Weaknesses
- Consider adding additional data collection tool and further question to telephone interview (See Approach weaknesses.)

1. Significance:
Strengths
- With many hospitals moving to electronic records and increasing electronic means of communication, an assessment of the impact on this changing environment is imperative. This project aims to do just that. With this information, issues can be identified with the potential to develop interventions to eliminate the negative impact that technology may foster.

Weaknesses
- Would like to see continued emphasis of identification of issues that various technologies have.

2. Investigators:
Strengths
- The PI has formed a strong group of investigators with previous experience in all areas needed for the success of this project.

Weaknesses

- None identified.

3. Innovation:

Strengths

- The rapidity of which hospitals are moving to electronic record systems makes this proposed project valid and innovative. This project would add to the paucity of information about the impact of EMRs.

Weaknesses

- None noted

4. Approach:

Strengths

- Conceptual framework is well articulated. The research environment is robust. Mixed methods approach is well-thought out. Ethnographic methods for gathering data for analysis are well documented.

Weaknesses

- To increase the likelihood of gaining more specific information about medication errors, consider adding to the telephone interview 4.c "To your knowledge, have communication practices between physicians and nurses ever caused any medical errors?" *If so please explain.*

- The investigators state that, "During shadowing, research assistants will collect information on education and experience for nurses; year of training and specialty for physicians; and all clinicians' experience with communication systems. Research assistants will also gather data from each nurse manager, physician leader, or other appropriate source (i.e., human resource records, medical training program administrator) on unit level demographic characteristics and information on the level of training for nurses and physicians who practice in each study unit." To maintain consistency of data collected, a demographic data sheet that details this information would be helpful. For instance "clinician's experience with communication systems"—is this all systems, length of time, what type of specific training. These are important variables that have the potential to influence results.

5. Environment:

Strengths

- Very favorable. Letters of support included.

Weaknesses

- None noted

Degree of responsiveness*:*

- No concerns

Budget and Period of Support*:*

- Seems appropriate for this project.

Protection of Human Subjects from Research Risks: Acceptable

Inclusion of Women and Minority Subjects: Acceptable

Inclusion of AHRQ Priority Populations*:* Acceptable

Resubmission Applications (formerly “revised/amended” applications):
- The PI has sufficiently addressed the concerns of the previous scientific review.

Resource Sharing Plan(s):
- The PI has sufficiently addressed the concerns of the previous scientific review.

Sharing Research Resources:
- The PI has sufficiently addressed the concerns of the previous scientific review.

CRITIQUE 3:

Significance:	2
Investigator(s):	2
Innovation:	2
Approach:	2
Environment:	2

Overall Impact:
Strengths
- Very important area to study
- Excellent investigator team
- Very well thought out research plan
- The reviewers have addressed the issues brought up by the first review

Weaknesses
- The dissemination plan could be stronger if the investigators agreed to publish in open-access journals and use an institutional repository to archive their work in.

1. Significance:
Strengths
- This work is highly significant given the unintended consequences surrounding health care provider communication occurring because of the adoption of new health information and communications technologies

Weaknesses
- None noted

2. Investigators:
Strengths
- The investigator team is strong and is likely capable to carry out a project of this complexity and magnitude as outlined in the application.

Weaknesses
- None noted

3. Innovation:
Strengths
- Having the physician members of the team attends the nursing focus group and the non-physician members attend the physician focus groups is innovative given that it allows team members to focus on communications issues from a new perspective.
- Using 4th year medical students to perform in hospital observation of physicians and nurses is innovative given they know the clinical environment that will allow them to be more unobtrusive

and interpret what is going on. Also performing observations in pairs is an excellent technique for improving accuracy and completeness of observations.

Weaknesses

- None noted

4. Approach:

Strengths

- The mixed-methods approach is a good choice for this extremely complex socio-technical study.
- The aims are well thought out and the use of the quantitative survey before the qualitative parts of this study are well justified.
- The investigator team has worked together in the past and has the benefit of experience from two pilot studies that directly inform the work outlined in the application.
- The theoretical framework is very well matched to this work.
- The categorization of using the HIMSS EHR Adoption model is an excellent way to systematically assess a given institutions state of HIT adoption.
- The sampling strategy for Aim 2 where there is stratification based on HIT Adoption level, Community versus teaching, and hospitalists versus non-hospitalist is a reasonable approach.
- Based on the size of membership of the NNPN and the letters of support from the NNPN director and four hospital systems, it is likely they will be able to meet the recruitment needs for Aim 3 (even though the letters from the hospitals are exact copies of one another.)

Weaknesses

- The dissemination plan would be improved if all the investigators agreed to publish in open-access peer-reviewed journals as the AHRQ does not participate in the NIH Public Access Policy
- The dissemination plan would also increase if the team would use an institutional repository to archive and make their scholarly output freely available in a form that is more consistent and perpetually available.

5. Environment:

Strengths

- The environment is supportive and adds significantly to the likelihood that the proposed work will be carried out successfully as outlined in the application.

Weaknesses

- None noted

Degree of responsiveness*:*

- The application is responsive to the FOA.

Budget and Period of Support*:*

- Overall, the budget is reasonable; however, there are some items that the AHRQ budgetary staff should address such as $500 for office furniture (file cabinet) and desktop PC's. These types of items should be covered by UM's 54% F&A.

Protection of Human Subjects from Research Risks: Acceptable

- There are adequate protections in place to protect the privacy of study subjects as well as the security of the data that will be collected during this work.

Inclusion of Women and Minority Subjects: Acceptable

- Women and minorities are included.

Inclusion of AHRQ Priority Populations: Acceptable

- This work focuses on how HIT and modern communications technologies affect physician and nursing communication. Therefore, this work will ultimately benefit all patient populations including AHRQ Priority Populations.

Resubmission Applications (formerly “revised/amended” applications):
- The resubmission is responsive to the previous reviewers’ issues.

CRITIQUE 4:

Significance: 2
Investigator(s): 2
Innovation: 2
Approach: 2
Environment: 2

Overall Impact:
Strengths
- Communication is critical to optimal care, and yet it is still not well understood
- This study addresses the FOA well
- It uses an innovative conceptual model, and addressing HIT in the context of nurse/physician communication is somewhat innovative
- Mixed methods are appropriate and suited to this study, and the personnel are qualified to do this work

Weaknesses
- Participation is key, and not completely guaranteed

1. Significance:
Strengths
- Communication is critical to optimal care, and yet it is still not well understood
- Study addresses FOA well
- Focusing on doctors and nurses makes a lot of sense

Weaknesses
- None noted

2. Investigators:
Strengths
- Strong group of researchers with the necessary skills to complete this work

Weaknesses
- None noted

3. Innovation:
Strengths
- Innovative conceptual model
- Addressing HIT in the context of nurse/physician communication is somewhat innovative

Weaknesses
- None noted

4. Approach:
Strengths
- Mixed methods are appropriate and suited to this study

- The personnel are qualified to do this work
- Survey methods are geared to generate participation
- Qualitative methods are well described
- Hospital buy in is more assured
- Potential problems are addressed

Weaknesses

- Participation is key, and not completely guaranteed

5. Environment:

Strengths

- Very well suited to complete this work

Weaknesses

- None noted

Degree of responsiveness*:*

- This proposal is very responsive

Budget and Period of Support*:* Appropriate

Protection of Human Subjects from Research Risks: Acceptable

Inclusion of Women and Minority Subjects: Acceptable

Inclusion of AHRQ Priority Populations*:* Acceptable

Resubmission Applications (formerly "revised/amended" applications):

- Responses are adequate and appreciated

THE FOLLOWING RESUME SECTIONS WERE PREPARED BY THE SCIENTIFIC REVIEW OFFICER TO SUMMARIZE THE OUTCOME OF DISCUSSIONS OF THE REVIEW COMMITTEE ON THE FOLLOWING ISSUES:

PROTECTION OF HUMAN SUBJECTS: ACCEPTABLE. There are adequate protections in place to protect the privacy of study subjects as well as the security of the data that will be collected during this work.

INCLUSION OF WOMEN PLAN: ACCEPTABLE. Women will be well represented in this study since they represent about 95% of all registered nurses.

INCLUSION OF MINORITIES PLAN: ACCEPTABLE. Minorities are included.

INCLUSION OF AHRQ PRIORITY POPULATIONS PLAN: ACCEPTABLE.

COMMITTEE BUDGET RECOMMENDATIONS: The budget was recommended as requested.

MEETING ROSTER

Health Care Quality and Effectiveness Research
Health Services Research Initial Review Group
AGENCY FOR HEALTHCARE RESEARCH AND QUALITY
HQER 1
October 28, 2013

CHAIRPERSON

CAREY, TIMOTHY S., MD, MPH.
PROFESSOR
DIRECTOR, CECIL G. SHEPS
CENTER FOR HEALTH SERVICES RESEARCH
UNIVERSITY OF NORTH CAROLINA
CHAPEL HILL, NC 27599

MEMBERS

BURGIO, LOUIS D., PHD
HRJ ENDOWED CHAIR
CENTER FOR MENTAL HEALTH AND AGING
UNIVERSITY OF ALABAMA
TUSCALOOSA, AL 35401

CARROLL, AARON E., MD *
PROFESSOR OF PEDIATRICS
ASSISTANT DEAN FOR RESEARCH MENTORING
DIRECTOR, CENTER FOR HEALTH POLICY AND PROFESSIONALISM RESEARCH
INDIANA UNIVERSITY SCHOOL OF MEDICINE
INDIANAPOLIS, IN 46202

DICOWDEN, MARIE A., PHD., FNAP *
EXECUTIVE DIRECTOR
BISCAYNE INSTITUTES OF HEALTH AND LIVING, INC
AFFILIATED WITH NEWYORK INSTITUTE OF TECHNOLOGY
NEW YORK COLLEGE OF OSTEOPATHIC MEDICINE
MIAMI, FL 33160

DILLARD, DENISE A., PHD *
RESEARCH DIRECTOR
RESEARCH DEPARTMENT
SOUTHCENTRAL FOUNDATION
ANCHORAGE, AK 99508

ESNAOLA, NESTOR F., MD, MPH, MBA, FACS *
PROFESSOR AND VICE CHAIR
CLINICAL AND ACADEMIC AFFAIRS; CHIEF,
DIVISION OF SURGICAL ONCOLOGY
DEPARTMENT OF SURGERY TEMPLE UNIVERSITY
SCHOOL OF MEDICINE
PHILADELPHIA, PA 19140

JACOBSON, ROBERT M., MD
PROFESSOR OF PEDIATRICS
RESEARCH CHAIR, EMPLOYEE AND COMMUNITY HEALTH
MAYO CLINIC
ROCHESTER, MN 559050001

KROTH, PHILIP J., MD *
ASSOCIATE PROFESSOR
HEALTH SCIENCES LIBRARY AND INFORMATICS CENTER
UNIVERSITY OF NEW MEXICO
ALBUQUERQUE, NM 87131

LINZER, MARK , MD *
DIVISION DIRECTOR
DIVISION OF GENERAL INTERNAL MEDICINE
HENNEPIN COUNTY MEDICAL CENTER
MINNEAPOLIS, MN 55415

MEHR, DAVID R., MD, MS
PROFESSOR
DEPARTMENT OF FAMILY AND COMMUNITY MEDICINE
SCHOOL OF MEDICINE
UNIVERSITY OF MISSOURI
COLUMBIA, MO 65212

MORIOKA-DOUGLAS, NANCY, MD, MPH.
CLINICAL PROFESSOR OF MEDICINE
CENTER FOR EDUCATION AND RESEARCH IN FAMILY AND COMMUNITY MEDICINE
CLINIC CHIEF, STANFORD FAMILY MEDICINE
STANFORD GERIATRIC EDUCATION CENTER
STANFORD, CA 94305

NAGYKALDI, ZSOLT J., PHD *
ASSOCIATE PROFESSOR
ASSOCIATE DIRECTOR OF RESEARCH
UNIVERSITY OF OKLAHOMA HEALTH SCIENCES CENTER
DEPARTMENT OF FAMILY AND PREVENTIVE MEDICINE
OKLAHOMA CITY, OK 73104

POSES, ROY M., MD
PRESDENT
FOUNDATION FOR INTEGRITY AND RESPONSIBILITY IN MEDICINE
WARREN, RI 02885

RAHMAN, MAHBOOB , MD
ASSOCIATE PROFESSOR
DEPARTMENT OF MEDICINE
CASE WESTERN RESERVE UNIVERSITY
CLEVELAND, OH 441064982

ROGERS, SELWYN O., MD
CHAIRMAN
DEPARTMENT OF SURGERY
TEMPLE UNIVERSITY
PHILADELPHIA, PA 19140

ROSENMAN, MARC B., MD *
ASSOCIATE PROFESSOR
DEPARTMENT OF PEDIATRICS
INDIANA UNIVERSITY SCHOOL OF MEDICINE
INDIANAPOLIS, IN 462025167

ROWAN, PAUL J. PHD., MPH
ASSISTANT PROFESSOR
SCHOOL OF PUBLIC HEALTH
DIVISION OF MANAGEMENT POLICY
AND COMMUNITY HEALTH
UNIVERSITY OF TEXAS-HOUSTON
HOUSTON, TX 77030

SCORDO, KRISTINE A., PHD *
PROFESSOR AND DIRECTOR ACUTE CARE NURSE PRACTITIONER PROGRAM
WRIGHT UNIVERSITY
DAYTON, OH 45435

SIMMONS, SANDRA F., PHD
ASSOCIATE PROFESSOR
VANDERBILT UNIVERSITY, SCHOOL OF MEDICINE
INSTITUTE OF MEDICINE AND PUBLIC HEALTH
CENTER FOR QUALITY AGING
NASHVILLE, TN 372322400

SINCLAIR, KA'IMI A., PHD, MPH *
ASSISTANT PROFESSOR
1730 MINOR AVE., SUITE 1760, SEATTLE, WA 98101
SEATTLE, WA 98101

SINGH, RANJIT, MBBCHIR, MD, MBA *
VICE CHAIR FOR RESEARCH
ASSOCIATE PROFESSOR OF FAMILY MEDICINE
ASSOCIATE DIRECTOR, PATIENT SAFETY RESEARCH CTR
STATE UNIVERSITY OF NEW YORK AT BUFFALO
BUFFALO, NY 14215

STEARNS, SALLY C., PHD
PROFESSOR
DEPARTMENT OF HEALTH POLICY AND MANAGEMENT
THE GILLINGS SCHOOL OF GLOBAL PUBLIC HEALTH
THE UNIVERSITY OF NORTH CAROLINA AT CHAPEL HILL
CHAPEL HILL, NC 27599

VAWDREY, DAVID K., PHD
ASSISTANT PROFESSOR
COLUMBIA UNIVERSITY
DEPARTMENT OF BIOMEDICAL INFORMATICS
NEW YORK, NY 10032

ZHANG, QI , PHD
ASSOCIATE PROFESSOR
SCHOOL OF COMMUNITY AND ENVIRONMENTAL HEALTH
OLD DOMINION UNIVERSITY
NORFOLK, VA 23529

ZORRILLA, CARMEN D., MD *
PROFESSOR
DEPARTMENT OF OBSTETRICS AND GYNECOLOGY
MATERNAL INFANT STUDIES CENTER
UNIVERSITY OF PUERTO RICO SCHOOL OF MEDICINE
RIO PIEDRAS, PR 00935

SCIENTIFIC REVIEW ADMINISTRATOR

PONCE-GONZALEZ, ILEANA M., CNC, MPH, MD
SCIENTIFIC REVIEW OFFICER
DIVISION OF SCIENTIFIC REVIEW (DSR)
OFFICE OF EXTRAMURAL RESEARCH, EDUCATION AND PRIORITY POPULATIONS (OREP)
AGENCY FOR HEALTHCARE RESEARCH AND QUALITY (AHRQ)
ROCKVILLE, MD 20850

GRANTS TECHNICAL ASSISTANT

ROBINSON, DIANA C.
PROGRAM ANALYST
DIVISION OF SCIENTIFIC REVIEW (DSR)
OFFICE OF EXTRAMURAL RESEARCH, EDUCATION AND PRIORITY POPULATIONS (OEREP)
AGENCY FOR HEALTHCARE RESEARCH AND QUALITY (AHRQ)
ROCKVILLE, MD 20850

* Temporary Member. For grant applications, temporary members may participate in the entire meeting or may review only selected applications as needed.

Consultants are required to absent themselves from the room during the review of any application if their presence would constitute or appear to constitute a conflict of interest.
